# Robotic Exoskeletons: A Perspective for the Rehabilitation of Arm Coordination in Stroke Patients

**DOI:** 10.3389/fnhum.2014.00947

**Published:** 2014-12-01

**Authors:** Nathanaël Jarrassé, Tommaso Proietti, Vincent Crocher, Johanna Robertson, Anis Sahbani, Guillaume Morel, Agnès Roby-Brami

**Affiliations:** ^1^UMR 7222, Center National de la Recherche Scientifique (CNRS), Institute of Intelligent Systems and Robotics (ISIR), Paris, France; ^2^UMR 7222, Sorbonne Universités, UPMC Univ Paris, Paris, France; ^3^U1150, Institut National de la Santé et de la Recherche Médicale (INSERM), Agathe-ISIR, Paris, France; ^4^Department of Electrical and Electronic Engineering, University of Melbourne, Melbourne, VIC, Australia; ^5^Department of Physical Medicine and Rehabilitation, Hôpital Raymond Poincaré, Garches, France

**Keywords:** rehabilitation robotics, exoskeleton, upper-limb, synergies, arm coordination control

## Abstract

Upper-limb impairment after stroke is caused by weakness, loss of individual joint control, spasticity, and abnormal synergies. Upper-limb movement frequently involves abnormal, stereotyped, and fixed synergies, likely related to the increased use of sub-cortical networks following the stroke. The flexible coordination of the shoulder and elbow joints is also disrupted. New methods for motor learning, based on the stimulation of activity-dependent neural plasticity have been developed. These include robots that can adaptively assist active movements and generate many movement repetitions. However, most of these robots only control the movement of the hand in space. The aim of the present text is to analyze the potential of robotic exoskeletons to specifically rehabilitate joint motion and particularly inter-joint coordination. First, a review of studies on upper-limb coordination in stroke patients is presented and the potential for recovery of coordination is examined. Second, issues relating to the mechanical design of exoskeletons and the transmission of constraints between the robotic and human limbs are discussed. The third section considers the development of different methods to control exoskeletons: existing rehabilitation devices and approaches to the control and rehabilitation of joint coordinations are then reviewed, along with preliminary clinical results available. Finally, perspectives and future strategies for the design of control mechanisms for rehabilitation exoskeletons are discussed.

## 1 Shoulder–Elbow Coordination and Synergies in Stroke Patients

Although stroke causes lesions of the motor areas of the brain, motor impairments occur in the body on the opposite side to the lesion (hemiplegia). During the weeks following the lesion, symptoms usually recover spontaneously but partially and inconstantly, and many patients are left with impairment of upper-limb movement (hemiparesis). The symptoms, which occur following a brain lesion, are classically termed negative symptoms (weakness (Colebatch and Gandevia, 1989; Sukal-Moulton et al., 2014), loss of individual joint control (Zackowski et al., 2004)) and positive symptoms with excessive muscle contractions (spasticity (Lance, 1980; Mottram et al., 2009), spastic co-contraction (Gracies, 2005), dystonia, or pathological synergies (Twitchell, 1951; Brunnstrom, 1970)). Together, the weakness and abnormal contractions result in a disruption of goal-directed upper-limb movements and hand dexterity, causing disability.

### 1.1 Synergies and shoulder–elbow coordination

Different practical and theoretical approaches are used to describe shoulder–elbow coordination. These descriptions are not fully consistent, with the word “synergy” being used to describe different phenomena. On one hand, Bernstein (1967) described synergies as fundamental building blocks of motor control, which decrease the redundancy of the system. Indeed, the motor system has more degrees of freedom (DoF) than are necessary to carry out any task, for example, the articulated upper-limb has more than 7 degrees of freedom when only 6 are necessary for any grasping task. According to this concept, synergies (i) combine several elements, which share the same spatio-temporal properties and “work together,” and (ii) may be combined in a task specific way so that a limited number of synergies can give rise to a continuum of responses. But, there is still no agreement on the space (muscle or joints) in which synergies are organized. For some authors, synergies are organized at the muscle level (Bizzi et al., 2008). Mathematical techniques such as linear decomposition have been used to identify muscle synergies in healthy subjects in a variety of tasks such as posture (Ting, 2007) or reaching (d’Avella et al., 2006, 2008). For other authors, synergies are organized at the joint level and are endowed with properties of flexibility and automatic compensation between elements in order to stabilize the important task related variable (Latash and Anson, 2006). On the other hand, the word synergy is also used to refer to the pathological coupling of movements observed in patients (Dewald and Beer, 2001). Clinical observations of the global and stereotyped patterns of movements that occur when stroke patients make any effort to move are described as *pathological synergies*. Couplings of shoulder elevation movements with elbow flexion (*flexor synergy*) or shoulder adduction/internal rotation with elbow extension (*extensor synergy*) have been reported (Brunnstrom, 1970). These abnormal synergies have been documented using quantitative experimental methods. Tasks involving the isometric generation of force have demonstrated that abnormal muscle coupling induces involuntary elbow flexion during voluntary shoulder abduction (Dewald et al., 1995; Dewald and Beer, 2001). During reaching movements in the horizontal plane at the shoulder level, target dependent perturbations of reaching kinematics and kinetics occur in stroke patients. These are reduced when the arm is supported against gravity (Beer et al., 2004; Sukal et al., 2007; Ellis et al., 2008). The available reaching workspace depends on the degree of shoulder loading (Sukal et al., 2007; Ellis et al., 2008). There are relatively few studies of muscle synergies using appropriate mathematical methods in hemiparetic patients. Cheung et al. (2009) showed that the structure of synergies was relatively preserved during various motor tasks in mildly affected patients but was either fractionated or merged in more impaired patients (Cheung et al., 2012). Using a 3D isometric task, Roh et al. (2013) did not find abnormal coupling between elbow and shoulder muscles but instead, abnormal and global activation of the three heads of the deltoid muscle during abduction.

Kinematic analysis of the time course of joint rotations showed a disruption of the relative timing between shoulder and elbow movements during reaching in stroke patients (Levin, 1996; Cirstea et al., 2003; van Kordelaar et al., 2012). During forward reaching movements, shoulder flexion and elbow extension tend to be reduced and shoulder abduction increased (Roby-Brami et al., 2003b). This elevates the elbow and alters the plane of arm movement (Merdler et al., 2013). The analysis of arm coordination is complicated by the fact that stroke patients develop compensatory trunk flexion (Cirstea and Levin, 2000; Roby-Brami et al., 2003a). A study based on principal component analysis (PCAs) showed that, when the trunk is fixed, patients with higher levels of impairment use fewer synergic joint combinations to carry out reaching tasks, suggesting that there is a reduction in the flexibility of synergies (Reisman and Scholz, 2003). Automatic error compensation between joint rotations is also impaired in stroke patients but appears to be task dependent since it is relatively preserved when the trunk is fixed (Reisman and Scholz, 2003) but not when trunk movement assists the reach (Reisman and Scholz, 2006). When the trunk is free, stroke patients use more synergic joint combinations than healthy subjects (van Kordelaar et al., 2012).

### 1.2 Mechanisms of altered joint coordination after stroke

The neurophysiology mechanisms behind synergies are still poorly understood. The specific role of the spinal cord (Bizzi et al., 2008), sub-cortical structures, and cortical areas (Cheney and Fetz, 1985; Capaday, 2004) in the generation of synergies is still unclear (i.e., in which structures of the hierarchical motor system are the elements gathered and the synergies combined?). Likewise, the relationship between voluntary control of individual joints, spasticity, and altered inter-joint coordination in stroke patients is still disputed. One current physiopathological hypothesis is that the abnormal fixed patterns are related to the activity of sub-cortical structures or networks. Owing to the alteration of the cortico-spinal command, the motor command is generated by less inhibited pathways, which originate in the brain-stem (Gracies, 2005; Cheung et al., 2009). This hypothesis is supported by the pattern of interaction of voluntary commands with multi-joint stretch reflexes (Trumbower et al., 2010), flexion reflexes (Dewald et al., 1999), the startle reflex (Honeycutt and Perreault, 2012), and neck rotation similar to the tonic neck reflex (Ellis et al., 2012). An increase in proprio-spinal relay is also likely (Mazevet et al., 2003). However, the alteration of the command, resulting from the lesion of the primary motor cortical area probably also has direct consequences on arm coordination. According to Zackowski et al. (2004), the main cause of impairment is the alteration of individual joint commands. Other authors have shown that reaching impairments are more related to a lack of recruitment of the agonists than to excessive coupling (Wagner et al., 2007; Prange et al., 2010). The abnormal coupling between joints could be a consequence of the distribution of muscle weakness and a saturation phenomenon (McCrea et al., 2005) (but this is contested by Beer et al. (2007)). The link between cortico-spinal command, spasticity, and coordination could be related to a deficit in the range of regulation of stretch reflex thresholds. The descending commands directly influence the level of excitability of the motoneuron membrane determining the position of the stretch reflex threshold relative to joint motion (Feldman et al., 2007). Impairments in the descending command modify its range of regulation causing spasticity (Levin and Dimov, 1997). Since spatial spasticity zones are modified by the shoulder–elbow configuration, this could be the basis of disordered upper-limb coordination (Musampa et al., 2007).

The mechanisms of recovery after stroke are multifactorial and the effect of rehabilitation programs is complex (Langhorne et al., 2011). Activity-dependent neural plasticity of the cortical maps adjacent to the lesion probably occurs, particularly during the acute period after stroke (Nudo, 2013). In order to stimulate such plasticity, many new rehabilitation methods, including robotic assistance, have been developed according to the principles of motor learning (Huang and Krakauer, 2009). In addition, the improvements measured by clinical scales can be due to the development of compensatory strategies. Compensatory strategies have an immediate benefit on daily life activity but, due to the learned disuse phenomenon, may have a negative impact on the quality of movement performance and limit the long-term prognosis (Taub et al., 2006; Levin et al., 2009). This is demonstrated by the possible benefits of constraint induced movement therapy (CIMT) and intensive task specific practice that can reduce learned disuse in the chronic stage of stroke (Wolf et al., 2006). After CIMT, some patients may continue to improve spontaneously if they have reached a given functional threshold so that they can use their limb spontaneously; if this is not the case, the benefit may be lost (Hidaka et al., 2012).

### 1.3 Robotic and mechanical assistance for recovery

Numerous rehabilitation-robotic devices have been developed since the late 90s, particularly for the neurorehabilitation of post-stroke patients (see review in Brewer et al. (2007)). Most of these devices guide the movement of the hand in one plane. Some robots can passively mobilize the limb of patients with poor recovery or can provide precisely controlled active assistance as a function of patient’s capacity. An advantage of robotic assistance is the possibility for patients to carry out a great number of movement repetitions, increasing the intensity of therapy. Recent extensive clinical testing of one of these devices, the InMotion^©^ robot (which has been used in clinical practice for many years) has demonstrated its effectiveness with significant improvements in arm motor capacity after a program of robot therapy. However, so far, there does not appear to be a qualitative benefit of robotic devices over a therapist performing the same quantity of movements (Volpe et al., 2008, 2009; Lo et al., 2010). Robot therapy still remains particularly interesting, however, since it affords more movement opportunities than standard therapy. Most previous clinical studies have been carried out with planar robotic manipulanda, which can only control the movement of the hand in space. In contrast, more conventional therapies (Brunnstrom, 1970; Bobath, 1990) particularly insist on the quality of the coordination based on the handling skills of the physical therapist. The physical therapist supports the weight of the upper limb by simultaneously holding the upper and the lower arm in order to mobilize the upper arm or to assist voluntary reaching movements. In addition, the support provided by the therapist is important for the prevention of shoulder–hand syndrome due to shoulder subluxation. A combination of these approaches would involve the insistence on the quality of coordination, monitored or guided by a therapist, while the patient practices a motor learning program (Levin and Panturin, 2011).

### 1.4 Can hemiparetic patients relearn coordination?

The ability of stroke patients to retain or relearn upper-limb coordination remains a topic of debate. The improvement of arm coordination observed during the acute phase of stroke is attributed to a spontaneous recovery of individual muscle commands (van Kordelaar et al., 2013). However, experiments performed in the chronic phase of stroke suggest that hemiparetic patients might not use all their potential shoulder–elbow coordination if they have the possibility to compensate by using the trunk. When the trunk is restrained, patients with moderate impairment show an immediate improvement in shoulder–elbow joint range and coordination (Michaelsen et al., 2001). Further studies have shown that repetitive training of reaching increases the amount of elbow joint rotation when the trunk is restrained but increases trunk compensation if it is free (Michaelsen et al., 2006), see review in Wee et al. (2014). This is consistent with the observation that CIMT does not improve joint range of motion (Kitago et al., 2013) since CIMT allows trunk compensation. Training the upper limb in the horizontal plane with gravity compensation using a robotic manipulandum (Dipietro et al., 2007; Tropea et al., 2013), progressive abduction loading (Ellis et al., 2009), or antigravity support (Krabben et al., 2012) improves the volume of the available workspace and the smoothness of the movement, suggesting that an improvement of arm coordination or a reduction of abnormal coupling. Other studies have not confirmed the benefit of such repetitive training on joint range of motion (Frisoli et al., 2012). A recent study suggested slight modification of muscle synergies after treatment (Tropea et al., 2013). Evidence that training not only improves active joint range of motion but also inter-joint coordination is still lacking.

## 2 Design Challenges for Rehabilitation Exoskeletons

While upper-limb manipulanda and lower limb exoskeletons have been used in the clinical practice for several years now (see the manipulandum by InMotion^©^ and the Lokomat^©^ by Hocoma), upper-limb exoskeletons have been only recently developed (mid 2000s) and their effects have been little studied. Indeed, the first commercially available upper-limb exoskeleton for rehabilitation was only released at the end of 2011 (Riener et al., 2011).

One major cause of this slow development is the complexity of the interaction between mechatronic structures and the human body, both at the physical and at the control level. While the pioneer devices only controlled hand motion in a single plane, exoskeletons provide 3D interaction at the joint level through their kinematic redundancy and the presence of multiple attachment points between the device and patient’s limb. These characteristics offer new and interesting perspectives for rehabilitation, but make devices much more complex to design and control.

Moreover, one fundamental particularity of rehabilitation exoskeletons compared to exoskeletons, which are designed to assist fully paralyzed patients, is that they should be able to respond to any movement made by the patient (even pathological). This must be based on a fine control of the mechanical interaction with the patient’s limb (Maclean et al., 2000): more than assisting the movement, the goal is to help the patient recover his/her sensorimotor capability. In order to be able to perform such a task, several challenges relating to the global mechanical design of these structures, their coupling with the human limb and, above all, their control, must be overcome.

### 2.1 Antagonistic design: Power vs backdriveability

From a mechanical point of view, an exoskeleton must be able to interact with the human body, a very complex kinematic structure. Exoskeletons must have a large number of active (motorized) joints, each with a wide range of motion to be able to follow as well as to assist movements within a large workspace. The exoskeleton must be able to both generate a high level of forces to sustain, assist, and/or perturb the motor capabilities of the patient, and to follow, without perturbing, human movements, which have large velocity and acceleration peaks, thus, requiring a high level of dynamic interaction. The main problem in designing such a device is the competing nature of these issues: a powerful exoskeleton requires large and heavy actuators; however, these limit angular displacement and therefore the workspace. Smaller, lighter actuators with gearboxes could generate sufficient forces, however, gearboxes add friction to the system, reducing overall dynamic performance. Innovative mechanical transmission combining high dynamics, forces, and ranges of velocities, which are compatible with human movement have been designed over the last 20 years, along with novel approaches to actuation (pneumatic, hydraulic, and cable actuators, combined, or not, with conventional electrical actuation) in order to adapt to the requirements for rehabilitation (Garrec et al., 2008). Thus, the challenge in the design of an exoskeleton is to achieve a “conceptual agreement” between power, workspace, dynamics, and weight.

### 2.2 Physical coupling between the human body and the robot and kinemato-static compatibility

**Figure 1:**
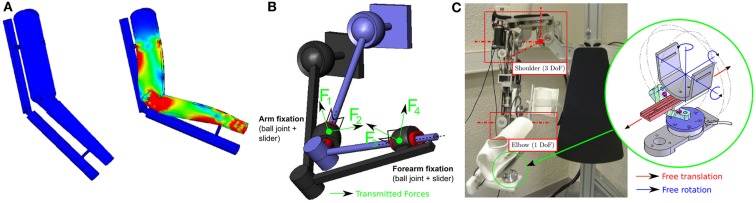
**(A)** Visualization of the strain distribution on a human arm performing a flexion movement when it is rigidly connected to a simple 1 DoF elbow exoskeleton with misaligned joint axes (Simulated with Solidworks^©^). Red areas represent the stress concentration zones. **(B)** Kinematic representation of a 4-DoF exoskeleton attached to a human arm (both with 3-DoF ball joint at the shoulder + 1 pivot joint at the elbow) using passive joint fixations (Jarrassé and Morel, 2012). **(C)** Four active DoF exoskeleton (ABLE, see Garrec et al. (2008)) with its set of passive DoF fixations.

For years, research has mainly focused on technological aspects (actuators, embedment, energy, etc.) and has followed the paradigm defined in Perry et al. (2007): “an exoskeleton is an external structural mechanism with joints and links corresponding to those of the human body.” In other words, designing the kinematics of an exoskeleton generally consists of trying to replicate human limb kinematics. This has a number of advantages: similarity of the workspaces, avoidance of singularity (Pons, 2008), and one-to-one mapping of joint force capabilities over the workspace. However, this paradigm has a major disadvantage due to the fact that it is impossible to precisely replicate human kinematics with a robot. Indeed, there are two major problems: morphology varies drastically between subjects and joint kinematics are very complex and cannot be imitated by conventional robotic joints (Scott and Winter, 1993). Currently, there is no consensual model of human kinematics in the biomechanics literature due to the complex geometry of bone surfaces. For example, different models are used for the shoulder complex including the scapula and clavicle (Van der Helm et al., 1992). Discrepancies between the two kinematic chains thus seem unavoidable, generating kinematic incompatibility and thus hyperstaticity or “over-constraint”: if the human and the robot are rigidly connected together through embedments at the different connection points, there will be more force and torque constraints than controlled mobility (active DoF) of the robot. The main consequence will be the occurrence of uncontrollable forces creating deformations of the subjects skin and tissues (Schiele, 2008) as can be seen in Figure 1A. This is even more problematic if the patient is fragile or has sensorimotor impairment.

Several approaches have been developed to avoid this problem. The first consists of designing an adaptable exoskeleton with adjustable length segments or the addition of redundant DoF serially in the robot kinematic chain to align active joint axes to the human joint axes (Housman et al., 2007). The other approach, based on the theory of mechanisms, involves the addition of passive DoF to connect the subject to the robot. Such a system allows the transmission of the robot-controlled forces while guaranteeing freedom of movement of the wearer and limiting the uncontrolled and undesired application of forces to his/her limb (Jarrassé and Morel, 2012; Galinski et al., 2013). An example of a fixation mechanism based on this approach is shown in Figure 1C: passive DoF fixations (each composed of a ball joint and a slider) are used to connect a human limb to a 4 active DoF (3 at the shoulder and 1 at the elbow) exoskeleton. Because of kinemato-static duality, the exoskeleton is only able to control 4 forces (shown in Figure 1B), which are the only forces, which are transmitted by these mechanisms (making the coupling isostatic). No torque (because of the rotation allowed by to the 3-DoF ball joint) or force (because of the 1 DoF linear slider) can be applied along the limb segment. Moreover, the system guarantees kinematic compatibility between the exoskeleton and the wearer without requiring an accurate alignment of joint axes.

### 2.3 Transparency and control sharing

The main objective of the research on the hardware design of exoskeletons is to maximize their interaction capacity.

Rehabilitation robotics began by using robots to passively mobilize patient’s limbs during the first stages of rehabilitation (*passive mode*) when the patient is unable to move alone. However, the effectiveness of such passive movements for stimulating motor recovery was limited (Lynch et al., 2005). In order to stimulate motor recovery, it is essential for rehabilitation robots to exhibit finer mechanisms of interaction: shared control of movement must be possible as soon as the patient has recovered a minimal amount of motor capacity (Patton and Mussa-Ivaldi, 2002; Hogan et al., 2006). Indeed, since neurorehabilitation addresses issues related to motor learning, the machines must allow patients to express whatever movement they can without hindering or suppressing their motor capability (Hogan and Krebs, 2004).

Therefore, one key feature that rehabilitation exoskeletons should exhibit is transparency: the robot must be able to “hide” if the patient is capable of making the movement without assistance. In this situation (called *active mode*, as the termis based on the patient’s activity), the robot is passive and thus must not perturb the patient’s movement (especially since human movements are often performed with low limb stiffness, and are very sensitive to perturbations (Gomi and Kawato, 1997)). Equally, if the robot is used as a measurement tool to record the patient’s movement, it must influence the movement as little as possible.

Despite efforts made to design backdrivable mechanical structures, most exoskeletons are not intrinsically transparent and rely on sensors and control algorithms to improve their interaction capacity and to make them behave “therapeutically.”

## 3 Control of Exoskeletons for Upper-Limb Rehabilitation

### 3.1 Existing active exoskeletons for rehabilitation

We carried out a review of active systems with 3-DoF or more (with a minimum of control on two upper-limb joints: shoulder, elbow, or wrist) and found 30 different exoskeleton prototypes for neurorehabilitation of the upper limbs. For this review, we searched *PubMed, ClinicalTrials, IEEE Xplore Digital Library, Science Direct*, and *Google Scholar* databases, using different combinations of the following keywords: “upper limb, robot, exoskeleton, rehabilitation, assisted, shoulder, elbow, wrist, arm, therapy, stroke, and training.” Passive “spring-like” structures and cable robots with multiple contact points were not considered in this study. A few multi-contact multi-robot active systems were, however, included since they interact at the joint level similarly to exoskeletons (Lo and Xie, 2012; Maciejasz et al., 2014), see Table 1.

**Table 1:** **Exoskeletons for upper-limb rehabilitation (3-DoF systems controlling at least two joints out of the shoulder-elbow–wrist chain)**.

Project name	First reference	DoF				Experiment with patients
		a	p	Type	pHRI
**SUPPORTED MOTION OF SHOULDER–ELBOW–WRIST**						
ARAMIS	Colizzi et al. (2009)	6	0	e	2-sfh	Pignolo et al. (2012) – c
ARMinIV	Nef et al. (2007)	7	0	e	ufh	Klamroth-Marganska et al. (2014) – c
ArmeoPower^©^^a^	Riener et al. (2011)	6	0	e	ufh	*(Used in several hospitals)*
ARMOR	Mayr et al. (2008)	8	4	e	2-ufuh	Mayr et al. (2008) – p
BONES + SUE	Klein et al. (2008)	6	0	p	ufh	Milot et al. (2013) – c
CADEN-7	Perry and Rosen (2006)	7	0	e	ufh	
ETS-MARSE	Rahman et al. (2010)	7	0	e	ufh	
EXO-UL7^b^	Yu et al. (2011)	7	0	e	2-ufh	Simkins et al. (2013) – c
IntelliArm	Zhang et al. (2007)	7	2	e	ufh	Ren et al. (2013) – p
NTUH-ARM	Tsai et al. (2010)	7	2	e	ufh	
Rupert IV	He et al. (2005)	5	0	p	sufh	Zhang et al. (2011) – p
SRE	Tsagarakis and Caldwell (2003)	7	0	p	fh	
SUEFUL 7	Gopura et al. (2009)	7	1	e	uffh	
**SUPPORTED MOTION OF SHOULDER–ELBOW**						
–	Moubarak et al. (2009)	4	0	e	uf	
ABLE	Garrec et al. (2008)	4	0	e	uf	Crocher et al. (2012) – p
CAREX	Brackbill et al. (2009)	5	0	e	suf	
L-Exos	Frisoli et al. (2005)	4	1	e	ufh	Frisoli et al. (2009) – p
LIMPACT	Stienen et al. (2008)	4	6	h	uuff	
MEDARM	Ball et al. (2007)	6	0	e	uf	
MGA	Carignan et al. (2005)	5	1	e	uh	
MULOS	Johnson et al. (2001)	5	0	e	uff	
Pneu-WREX	Sanchez et al. (2005)	4	0	p	ufh	Reinkensmeyer et al. (2012) – c
RehabExos	Vertechy et al. (2009)	4	1	e	ufh	
**SUPPORTED MOTION OF ELBOW–WRIST**						
MAHI EXO-II	Gupta and O’Malley (2006)	5	0	e	ufh	*(Ongoing)* – c
MAS	Ding et al. (2008)	4	0	p	ufh	
ULERD	Song et al. (2014)	3	4	e	ufh	
**MULTI-CONTACT MULTI-ROBOT SYSTEMS**						
–	Morales et al. (2011)	6	0	p	uh	
iPAM	Culmer et al. (2005)	6	0	p	uf	Culmer et al. (2011) – p
NeReBot	Rosati et al. (2005)	5	0	e	fh	Masiero et al. (2013) – c
Reharob	Toth et al. (2004)	12^c^	0	e	uf	Fazekas et al. (2007) – c

*Number of degrees of freedom (DoF): a, active thus actuated; p, passive thus mechanical only. Type refers to the actuation system: e, electrical; p, pneumatic; h, hydraulic. Physical Human-Robot Interface (fixation levels): 2-two arm exoskeleton, s, shoulder; u, upper arm; f, forearm; h, handle. Double letters indicates double interfaces. Experiments: c, clinical; p, pre-clinical test. The last four projects are not strictly exoskeletons but rather multi-contact multi-robot systems*.

*^a^Based on ARMinII, the only commercialized exoskeleton for the clinical environment*.

*^b^Based on CADEN-7*.

*^c^Two 6-DoF industrial serial manipulators*.

Most exoskeletons found were designed to affect either the shoulder and elbow joints or the shoulder, elbow, and wrist. To our knowledge, only 11 of these 30 devices have been tested on post-stroke subjects, and few studies involved more than 10 post-stroke patients. About one-third of the devices found have not been the subject of publications in the main journals and conferences for at least 3 years.

### 3.2 Challenges in the control of an exoskeleton

**Figure 2:**
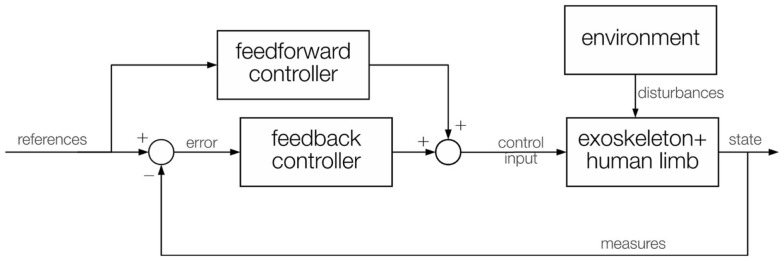
**General control scheme, with a feedback control that calculates the error and provides a control input, and a feedforward control that directly contains the desired values and provides another control input**. Both inputs are combined into a single control fed to the exoskeleton actuators. Measures of the current state are fed back to the controller. The interaction with the environment acts like a disturbance on the exoskeleton control algorithm.

Existing controllers for exoskeletons are mostly *assistive*, according to the definition in Marchal-Crespo and Reinkensmeyer (2009): this involves replicating therapist behavior to assist the impaired subject to accomplish specific tasks. This paradigm can be implemented using different control techniques. The simplest way to control any robotic device is to create a *feedback* loop (Figure 2): this closed-loop structure usually regulates the position or the interaction forces along a known reference (for example, a trajectory or a force field model) and can be applied either at the joint (exoskeleton) or at the end-effector level (manipulandum). This is often combined with *feedforward* control to compensate predictable perturbations in an anticipatory way (for example, the weight or the dynamics of the exoskeleton, and the friction forces within the joints).

Rigid position-feedback controllers combined with feedforward control are commonly used in exoskeletons (Johnson et al., 2001; Nef et al., 2007; Vertechy et al., 2009; Moubarak et al., 2010; Morales et al., 2011; Song et al., 2014). The feedforward compensation can be derived from the robot model, if available, or, since modeling the exoskeleton-human limb system is often complex, it can be learnt by using an adaptive control technique, by setting up a neural network (Yu and Rosen, 2013), or by iterative learning (Balasubramanian et al., 2008). The overall idea behind these methods is that, on the first trial no feedforward compensation is present and the feedback control must compensate for the entire error. Then, while the feedforward term is learnt trial-by-trial and increases, input from the feedback decreases, leading to better recovery from errors, in less time, with less oscillations and less rigidity on the human limb.

Feedback position controllers are sufficient for *fully assistive* modes of control (also known as *passive mode* or *rigid control*) or even for simple *active mode* control such as *assistance-as-needed* algorithms based on virtual guides. For example, Guidali et al. (2011) developed a virtual channel in which the subject moves: once he/she goes out of this channel, the feedback control returns him/her to it, as if a spring was attached from the limb to the center of the virtual channel. In addition, to prevent the subject from getting stuck during the motion, a supporting force in the direction of the channel was added and adapted depending on the mean velocity of the limb. A similar idea was used by Mao and Agrawal (2012) with a cable-driven system, a low-level feedback controller and the addition of feedforward control to control the cable tension. In the study by Wolbrecht et al. (2008), the authors implemented an *adaptive assisted-as-needed* controller, in which a feedback controller works with a feedforward assistive term, which is adapted during the motion depending on the dynamics of the patient’s limb, his/her neurological ability, and the effort he/she makes. Assistance-as-needed paradigms seem more suited for rehabilitation because the subject is pushed to make an effort to achieve the motion task. This is the key to retraining movement following stroke (Collantes et al., 2012; Pennycott et al., 2012; Krishnan et al., 2013).

A good compromise between tracking skills and the stiffness of the robot can be obtained using *impedance control* (Hogan, 1984). Impedance control can be seen as a force controller with position-feedback. The idea behind this controller is to regulate the relationship between tracking capacity and the rigidity of the robot by tuning the so-called *mechanical impedance Z*:

(1) $$\frac{F\left(s\right)}{X\left(s\right)}=sZ\left(s\right)$$

where *F*(*s*) is the force at the interface, *X*(*s*) is the output position, and *s* refers to the Laplace transformation. The more *Z* increases, the better *X* is tracked, the higher *F* is produced, and vice versa. The model of mechanical impedance *Z* is given by the classical mass-spring-damper model, where the proportional gain is the spring effect, the derivative is represented by the damping factor, and the inertial mass acts as an integrator. The aim is to match this virtual model to the real interaction between the robot, the human limb, and the environment. Thus, it is a model-based method of control. This controller inverse method is *admittance control*, which is a position control with feedback on the force. The force-tracking trade-off feature, together with the simplicity of implementation, are the reasons why these are two of the most common control algorithms currently used for rehabilitation exoskeletons (Gupta and O’Malley, 2006; Caldwell et al., 2007; Carignan et al., 2007; Frisoli et al., 2009; Culmer et al., 2010; Yu et al., 2011). Impedance control is efficient for lightweight backdrivable exoskeletons, in which cable-driven systems are often used for torque transmission. The problems relating to this type of control are the compensation of gravity and friction, particularly in tendon-like systems. For exoskeletons that lack backdrivability, admittance control may be more appropriate, because there must be measurements of the force at the interfaces with the human limb to move the robot, considering its inertia and dynamic effects.

Several research groups have attempted to use other, more complex forms of control. For example, Rahman et al. (2013) developed the *Sliding Mode Control with Exponential Reaching Law (SMERL)*, a non-linear control mode in which the tracking problem is projected onto the state space where a *sliding surface*, containing the reference trajectory, is derived. The aim of the controller is to constrain the exoskeleton motion onto this surface.

Other complex approaches are based on tuning the behavior of the robot to the patient’s action detected through *sEMG* or even *EEG* sensors. For example, Tsai et al. (2010) used an EMG-based trigger to inhibit a compensation term in an impedance controller if the electrical signal from the impaired muscles was large enough. Gopura et al. (2009) developed a neuro-fuzzy controller to determine a set of thresholds and thus a set of different levels of control shared between the subject and the exoskeleton via the impedance controller, based on the subject sEMG activity. In Ding et al. (2008), after an offline estimation of muscle force using motion capture and EMG sensors, assistive torques were provided by pneumatic actuators to the impaired limb when the human joint torque was insufficient to complete the movement. In Loconsole et al. (2014), sEMG sensors were used to estimate and predict the resulting joint torques. Using this prediction, a reference position trajectory was computed and fed to a feedback controller with gravity and friction compensation. Blank et al. (2013) augmented the physical human-robot interface with a non-invasive brain-machine interface. This approach brings the human being into the control loop by capturing his/her intention of movement and anticipating the assistance required. The main drawback with all these methods is the use of sEMG/EEG to detect specific events since these sensors lack accuracy. For example, there is a poor signal to noise ratio, there can be cross-talk from other muscles, and sweat from the patient may interfere with transmission.

### 3.3 Consideration of joint coordination in the exoskeleton control

#### 3.3.1 The problem of the reference trajectory

Most rehabilitation exoskeletons use a reference trajectory, i.e., a path and an associated velocity profile that the robot should follow. It is important to realize that the reference used by these controllers is a set of joint trajectories and not a model of joint coordination. Movements of intermediate joints either occur as a consequence of the end-effector movement in the task space, or are simply constrained along specific dedicated trajectories, which are synchronized within the joint space. These approaches thus provide simple and local solutions to the coordination problem but they do not directly address the coordination issue as a whole. Simply controlling all the joints of an exoskeleton does not address the problem of inter-joint coordination.

Defining the set of joint trajectories is also a major issue. Joint trajectories can be copied from recordings of movements in healthy subjects, informed by a therapist, or computed by an optimal trajectory planner, which, for example, relies on constraints placed on the swivel angle (around the line joining the shoulder to the wrist) to solve the redundancy of the exoskeleton and generate a suitable set of reference joint trajectories for the desired end-effector movement. However, relying on such input limits the efficacy of the control algorithms because these trajectories are generally position and time dependent and are therefore complex to generalize for different movements, targets, or tasks. This means that the patient’s freedom of movement with the exoskeleton is limited as coordination patterns can only be programed for specific movements. Essentially, exoskeleton platforms are therefore reduced to simple manipulanda in 3D space.

#### 3.3.2 Coordination of joint torque

**Figure 3:**
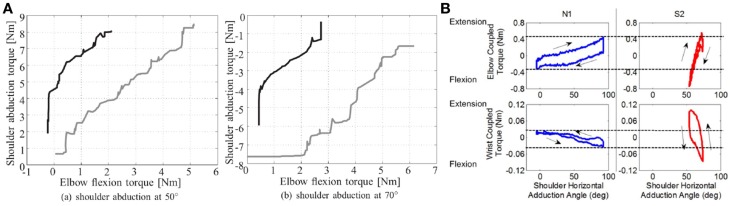
**Examples of experiments addressing joint torque coordination**. **(A)**. Decrease in the flexor synergy of one stroke patient: there was a decrease in involuntary elbow torque at 50° and 70° shoulder abduction at the end of the therapy. The gray lines show the torque before therapy and the black lines after (Guidali et al., 2011). **(B)**. Measures of elbow and wrist coupled torques as a function of the shoulder abduction angle for a healthy subject (N1) and a stroke survivor (S2) (Ren et al., 2013).

Guidali et al. (2009) developed an approach based on correction of the pathological involuntary flexion torque, which occurs at the elbow during shoulder abduction. During an evaluation session with the robot, the patient’s pathological involuntary torque was measured and a counter-active, just-as-needed torque was then calculated and applied during the therapy. A case study on one patient showed a reduction of elbow involuntary elbow flexor torque during shoulder abduction (Figure 3A) after 4 weeks of training (1.5 h/day, 3 days/week). Nevertheless, since passing from joint torque to joint kinematics is not simple and direct (because of the modulation joint stiffness modulation, among other issues), such an approach can not fully guarantee a positive and controlled effect on patient joint kinematic relationships.

Ren et al. (2013) used a similar approach with the IntelliArm exoskeleton to develop a passive stretching controller for multiple joints: individual joints are passively stretched by the robot in order to identify their individual angle-resistance torque relationships (an example of these relationships for healthy subjects and patient are shown in Figure 3B). These relationships are then used to coordinate the passive stretching of multiple joints together. Feasibility tests performed in 3 stroke patients showed a reduction of cross-coupled stiffness after a 40 min stretching session. However, no active modes of therapy (i.e., during which the patient is actively participating in the movement), based on previously identified angle-torque relationships, have been developed to target patterns of inter-joint coordination.

#### 3.3.3 Joint kinematic coordination

**Figure 4:**
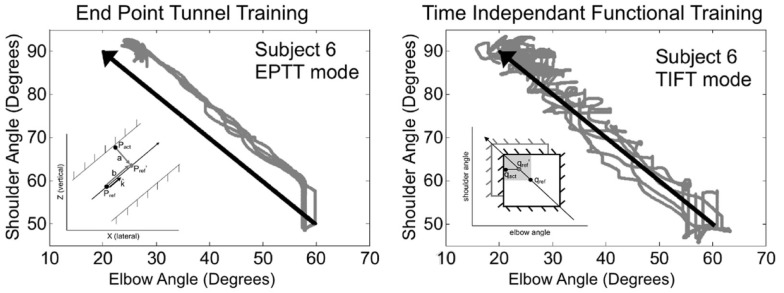
**Comparison of typical training trajectories from one stroke patient obtained with conventional end-point tunnel control (EPTT) and time independent functional training (TIFT) (Brokaw et al., 2013)**. In TIFT, subjects’ trajectories tend to be close to the ideal desired path. Illustrations of the principle of the movement constraints are shown within each figure (EPTT imposes constraints at the cartesien end-effector level while TIFT imposes them at the joint level).

Very few approaches have attempted to address the temporal and/or spatial relationships between joints. One approach, used with the ARMin III robot, is based on a time-independent functional training (TIFT) algorithm (Brokaw et al., 2013). This controller generates virtual joint-space walls to keep the subject close to the ideal joint-space path, acting both on multiple joint motion and feedback position control. Independence from time is important in order to allow the subject to actively achieve the task, without being constrained by rigid, planned trajectories. This strategy corrects undesirable coordination patterns between the shoulder and elbow joints. The main issue is once again the position-dependency requiring different reference paths for each joint and for each different motion. The performance of this control approach was tested on 10 moderate to severely impaired individuals with chronic stroke, and compared with results obtained with a conventional end-point tunnel algorithm (EPTT). Larger improvements in inter-joint coordination were obtained with the TIFT approach. Figure 4 shows examples of joint coordinations obtained for one subject with both control approaches, along with a representation of associated control approaches.

An alternative approach has been presented in Crocher et al. (2012). In this work, the problem of tracking is projected from the reference trajectory space to the joint velocities synergy space. The exoskeleton generates reactive viscous joint torques to impose specific patterns of inter-joint coordination without constraining hand motion.

For this approach, a first step consists of characterizing the inter-joint coordination required to achieve a given task. This characterization is performed using principal component analysis (PCA) in the joint velocity space. This technique is widely used in the study of synergies. PCA provides a different approach to the expression of joint velocity space by summarizing the *n_r_* (the number of considered joints) dimensions through principal component vectors **p**_1_ to $p_{n_{r}}$. Since the human arm has a redundant number of DoF for the performance of all common tasks (e.g., a reaching task in 3D requires only 3-DoF since the orientation is not constrained), only *m* < *n_r_* first principal components are required to fully represent the task to perform. The remaining *n_r_* − *m* components thus represent the unnecessary coordinations for the given task. The pathological synergies, or abnormal coordination, thus, “lie within” these latest components: $\left[p_{m+1}\ldots {\text{p}}_{{\text{n}}_{\text{r}}}]\right.$, which are used to define a movement constraint matrix $C\in R^{\left(n_{r}-m\right)\times n_{r}}$:

(2) $$C=\left[\begin{array}{c} p_{m+1}\\ \vdots \\ p_{n_{r}} \end{array}\right]=\left[\begin{array}{c} p_{4}\\ \vdots \\ p_{n_{r}} \end{array}\right](for\;a\;3\;DoF\;task)$$

A robotics control method is then used to constrain the movement outwith this unnecessary subspace such that at any given time instant:

(3) $$C{\overset{•}{q}}_{r}=0$$

where ${\overset{•}{q}}_{r}\in R^{n_{r}}$ is the joint velocity vector. The subject should thus produce a well coordinated movement with a velocity vector respecting the given constraint **C**. A correction is calculated for any velocity vector outside of the subspace necessary to achieve the task with the defined coordination. The corrective torque is a viscous field bringing the movement back into this desired movement subspace:

(4) $$\tau_{c}=-bC^{+}C{\overset{•}{q}}_{r}$$

where *b* is a scalar viscosity coefficient tuning the intensity of the correction. The exoskeleton torques τ_c_ will be null when the subject’s movement respects the defined coordination and will correct the movement without explicitly affecting the hand movement otherwise.

This approach was first experimented on a 4 DoF upper-limb exoskeleton (ABLE exoskeleton – a transparent screw-cable mechanical transmission based device, with 3-DoF for the shoulder and 1 for the elbow developed by CEA-LIST (Garrec et al., 2008) with healthy subjects performing 3-dimensional reaching tasks and showed its ability to impose un-natural synergies on healthy subjects without altering hand motion (Crocher et al., 2011).

**Figure 5:**
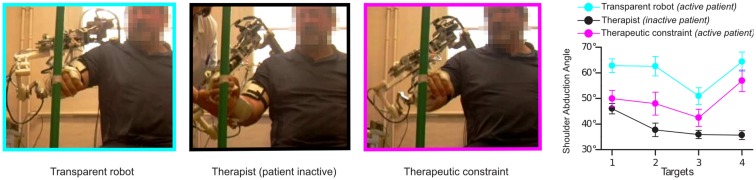
**The effect of the application of viscous constraints in the joint space**. On the left, one patient pointing to one of the four targets in several modes: with robot in transparent mode (no correction applied), with a therapist imposing the movement to the robot + arm (with the robot in a transparent mode) and with robot applying the therapeutic constraint (Crocher et al., 2012). Right: final abduction angle of robot + arm for each target (external, middle, internal, and high) averaged for all patients, measured in the different modes.

Preliminary experiments were also conducted with hemiparetic patients: before carrying out the task, a therapist passively guided the patient’s arm (wearing the robot in transparent mode) toward the targets, ensuring that the joints followed a normal pattern of coordination. This pattern of coordination was then used to define the matrix C of the active-constrained synergy-based controller. The results showed that the controller could impose constraints in the same way a therapist would do. In other words, the exoskeleton shaped the patient’s movement similarly to the therapist. As can be seen in Figure 5, the therapeutic constraint generated by the controller decreased shoulder abduction (part of an abnormal synergy), without significantly modifying the endpoint trajectory. This improvement in the upper-limb synergy while preserving the patient’s movement intention is a common rehabilitation goal. These preliminary studies, however, did not involve enough movement repetition or measures of post-effects to evaluate any learning effects or to make conclusions regarding the effect of this novel form of rehabilitation compared to conventional therapy.

## 4 Discussion and Perspectives

Therapy with exoskeletons can theoretically combine motor learning principles, which insist on the importance of intensive therapy using active movements, and the more classical methods, which are based on improving the quality of coordination. Some simple technological rehabilitation devices have recently been developed for this purpose. Most focus on one joint or a local group of joints such as hand or wrist rehabilitation robots or simple hand motion analysis systems (like inertial measurement units (IMU) or the Nintendo Wii^©^). These devices are, however, rarely functional and may lead the patient to develop compensatory strategies.

The use of a multi-contact robotic device for the rehabilitation of coordination thus seems promising. However, as explained in this paper, the development of exoskeletons for rehabilitation is only beginning and numerous technological, physiological, and clinical challenges lie ahead. With regard to the results of clinical investigations, the few studies, which have directly addressed the temporal and spatial relationships between joints have only been preliminary, involving a limited number of patients (listed in Section 3.3). Only the ARMin IV exoskeleton (Klamroth-Marganska et al., 2014) has so far been tested in a randomized controlled clinical trial. However, this study did not directly address coordination since the control used was the classical “assist-as-needed” method, along a pre-defined set of joint trajectories. The patients underwent 45 min of robot therapy, 3 times a week for 8 weeks during the chronic stage of stroke (>6 months) and the results showed a statistically significant improvement of the Fugl-Meyer score, however, the improvement was not clinically significant. In addition the benefit was not maintained at follow-up. The strength was not modified by therapy. This result is similar to a clinical trial involving the TWrex system (commercial name: Armeo Spring^©^), which is not motorized but uses a system of springs to assist arm movement (Housman et al., 2009). The relatively disappointing results of these two studies might be due to the chronic stage of the hemiparesis and/or to the limited duration of the therapy.

The current lack of positive results is not surprising, for several reasons. First, only a few devices currently have the high level of transparency, which is essential for the rehabilitation of inter-joint coordination with smooth corrective mechanisms similar to clinical practice. During Bobath therapy, the patient is active, occasionally guided or corrected, but not constantly constrained to follow a fixed gesture. In contrast, poorly transparent robots impose (more or less rigidly) constant constraints with pre-defined coordination or tunneling. This mode of interaction is less physiological and its efficiency is questionable.

The second reason is the lack of simple tools and metrics to assess inter-joint coordination. The clinical scales commonly used may not have been sensitive enough to evaluate specific improvements. More precise quantitative methods may be more adapted to evaluate changes and follow-up (Jarrasse et al., 2010; Kim et al., 2013), to specify indications for such therapy and later to determine the optimal dose and duration. Finally, the control of the redundant, multi-joint limbs by the CNS is still poorly understood (Guigon et al., 2007; Martin et al., 2009). It is therefore more difficult to incorporate an automatized generator of joint coordination trajectories in exoskeletons than it is to implement “human-like” end-effector trajectories (based, for example, on the minimum jerk theory) in manipulanda, since no consensual and simple model of redundancy resolution is available.

Indeed, the number of studies on the effect of perturbative or assistive fields at the inter-joint level is limited. Most experiments on physical interaction have been performed using planar manipulanda with 2D force fields and a single point of attachment between the subject and the robot. Translation of knowledge and methods from the research to the clinical environment was therefore simple for manipulanda, as both used similar platforms. Exoskeletons are still little used for research in motor control, despite the fact that it is critical to generalize existing motor control theories to a more complex framework including 3D force fields acting at the joint level with multiple interaction points. Studies of the after-effects, which occur following the application of joint constraints with an exoskeleton has, for example, never been studied in healthy subjects. Advances in these scientific fields should have an important impact on clinical exoskeletons, leading to the design of innovative approaches to rehabilitation with the training of inter-joint coordination. This improvement at the impairment level should limit the development of compensatory strategies, helping patients to reach their full functional potential (Taub et al., 2006; Levin et al., 2009).

## Author Contributions

Nathanael Jarrassé, Tommaso Proietti, Vincent Crocher, and Agnes Roby-Brami wrote the paper. Nathanael Jarrassé and Guillaume Morel authored the work presented in Section 2.2. Vincent Crocher, Johanna Robertson, Anis Sahbani, Agnes Roby-Brami, and Guillaume Morel authored the work presented in Section 3.4. Johanna Robertson and Agnes Roby-Brami carried out the related clinical experiments. All authors read and approved the submitted manuscript.

## Conflict of Interest Statement

The authors declare that the research was conducted in the absence of any commercial or financial relationships that could be construed as a potential conflict of interest.
